# R21/MATRIX malaria vaccine: awareness, perception, acceptability, and willingness to pay among caregivers of under-five children in endemic communities in Lagos, Nigeria

**DOI:** 10.1186/s12936-026-05803-5

**Published:** 2026-02-05

**Authors:** Progress Agboola, Danladi Nengak Precious, Yusuff Adebayo Adebisi, Obeta Odinaka Kingsley, Peter Olaniyi, Ayodele Ezekiel, Oto Peter Ode, Boluwatife Aderounmu, Agboola Peace Olusogo, Fawole Israel Opeyemi, Oluwafikayo S. Adeyemi-Benson

**Affiliations:** 1 Department of Medicine and Surgery, Ladoke Akintola University of Technology, Ogbomosho, Oyo State Nigeria; 2 Genomics and Research Department, Nigeria Centre for Disease Control, Abuja, Nigeria; 3 Systematic Reviews Network Akai Efa, Calabar, Cross River State Nigeria; 4 College of Social Sciences, University of Glasgow, Glasgow, UK; 5 Global Health and Infectious Disease Control Institute, Nassarawa State University, Keffi, Nigeria; 6 Medical Laboratory Science, Ladoke Akintola University of Technology, Ogbomoso, Nigeria; 7 Department of Public Health, First Moscow State Medical University, Moscow, Russia; 8 John Hopkins Bloomberg School of Public Health, Baltimore, MD USA; 9 Department of Nursing Science, Seventh Day Adventist College of Nursing, Ile-Ife, Osun State Nigeria; 10 Lead City University, Ibadan, Nigeria; 11 Department of Kinesiology and Community Health, University of Illinois at Urbana-Champaign, Champaign, IL USA

**Keywords:** Malaria, R21/MATRIX vaccine, Vaccine awareness, Vaccine acceptability, Willingness to pay, Caregivers, Lagos

## Abstract

**Background:**

Malaria remains a leading cause of morbidity and mortality, particularly among children under five years of age in sub-Saharan Africa. Nigeria has the highest burden of malaria cases worldwide, accounting for 26% of all cases. The introduction of the R21/Matrix malaria vaccine is promising for reducing the malaria burden. However, vaccine awareness, perceptions, and willingness to pay among caregivers are key to ensuring successful rollout. This study assessed caregivers' awareness, perceptions, acceptability, and willingness to pay for the R21/MATRIX malaria vaccine in endemic communities in Lagos, Nigeria.

**Methods:**

A community-based cross-sectional survey was conducted among 372 caregivers of children under five years of age. A multistage sampling technique was employed to select respondents from five malaria-endemic communities in Lagos. Data were collected via a semi-structured, interviewer-administered questionnaire. Descriptive statistics were computed, and binary logistic regression was used to identify predictors of vaccine awareness, perception, and willingness to pay.

**Results:**

Awareness of the R21/MATRIX vaccine was low (9.7%), with the internet as the primary source of information. Positive perceptions of the vaccine were high, with 98.1% of caregivers believing it to be safe and effective. Acceptability was also high, as 76.6% of the caregivers indicated that they would "definitely" vaccinate their child. Willingness to pay was reported by 71.2% of the respondents, with 51.9% willing to pay between ₦1,001–₦5,000 per dose. The factors significantly associated with awareness included gender, marital status, and relationship with the child (p < 0.05). Higher household income and guardianship status increased the likelihood of willingness to pay (p < 0.05).

**Conclusions:**

Despite the low awareness of the R21/MATRIX malaria vaccine, caregivers demonstrated a strong positive perception, high acceptability, and moderate willingness to pay. Targeted community awareness campaigns focused on the benefits and safety of the vaccine are essential to improve knowledge of the vaccine in endemic communities.

## Background

Malaria remains a global health challenge, as approximately 263 million estimated malaria cases were reported globally in 2023 in 83 malaria-endemic countries. This figure is 11 million cases higher than that in 2022 [1]. Among children under five years of age in sub-Saharan Africa, malaria remains the leading cause of morbidity and mortality [2]. In the African region, there were 246 million cases and 569,000 deaths in 2023, with 76% of deaths in the area being among children under 5 years of age. Additionally, in 2023, Nigeria (26%) had the highest estimated burden of malaria cases [1]. In Lagos state, malaria has been reported to be high in different regions including Ikorodu's Ibeshe community (14.7%) [3] and Makoko slum (21.1%), making them malaria endemic communities [4].

Among children under five years of age in Nigeria, Malaria is a leading cause of global child mortality, with 95,000 annual child deaths [5]. This is largely due to their underdeveloped immune system, which is unable to produce sufficient antibodies to combat Plasmodium parasites, increasing their vulnerability [6]. As a result, the consequences could be extremely harmful and possibly lead to seizures, coma, and anemia from recurrent infections, all of which increase the child's risk of death [2].

Malaria also has many economic and social consequences in endemic communities, including impeding children's education, reducing workers' productivity (which in turn affects family income and livelihood), increasing out-of-pocket expenses, and causing the Gross Domestic Product (GDP) to grow 1.3% less per person annually [7]. In Nigeria, many factors increase the risk and prevalence of malaria infection, such as the climatic conditions, spatial ecology and behavioral dynamics of Anopheles mosquitoes, social and economic factors, and existing malaria prevention and management programs [8]. Specific risks for children under five years of age include low levels of educational attainment among mothers [9], child age, lack of use of mosquito bed nets, limited access to medical facilities, unsafe sources of drinking water, and living far from waste disposal sites [10].

Efforts to combat malaria in Nigeria have been extensive. The 2013 National Malaria Strategic Plan (NMSP) initiative, which aimed to eliminate malaria as a public health burden in the country, was established to run for seven years from 2014–2020, but the target was unmet. This led to the development of a new strategic plan for 2021–2025, which aims to reduce malaria-related mortality to fewer than 50 deaths per 1,000 live births and achieve a prevalence below 10% by 2025 [10, 11]. The Nigerian End Malaria Council (NEMC) was established in 2022 to strengthen malaria elimination efforts by 2030 [12]. Currently, Nigeria uses different malaria preventive intervention targeting the under 5 population, including insecticide-treated nets (ITNs), indoor residual spraying (IRS), larval source management (LSM), antimalarial drugs, and intermittent preventive treatment in pregnancy (IPTp) [11]. However, significant challenges persist, such as drug and insecticide resistance, insufficient ITN and IRS coverage, and gaps in diagnostic testing [11].

The development of vaccines against malaria is promising for addressing these challenges. The World Health Organization (WHO) approved the RTS, S/AS01, and R21/Matrix-M vaccines in 2021 and 2023, respectively, to prevent malaria globally [13]. Recent measures in Nigeria include the approval of the R21/MATRIX malaria vaccine in 2023. The R21 vaccine, with up to 80% efficacy, offers hope as a scalable alternative to the RTS, S vaccine, which may not be available owing to manufacturing and funding constraints [11]. Nigeria has acquired one million doses of the R21 malaria vaccine, with 846,200 doses funded by GAVI, the Vaccine Alliance, and 153,800 doses purchased by the Nigerian federal government. Vaccine rollout implementation prioritized Bayelsa State in southern and Kebbi State in northern Nigeria because of their high malaria burdens [14]. The National Primary Healthcare Development Agency (NPHCDA) has integrated the vaccine into routine immunization programs, which target children aged five to fifteen months [14]. The vaccine is given as a three-dose intramuscular series with a booster after one year and provides higher efficacy, a more consistent immune response, and greater potential for herd immunity compared with other interventions, complementing malaria control and reducing disease burden in children [15].

The WHO reports that at $2 to $4 per dose, the R21 malaria vaccine has the potential to be a cost-effective intervention against malaria [16]. While this approach is promising, perception, acceptance, and potential uptake remain major obstacles to achieving herd immunity [17]. In a study by Ajayi and Emeto (2023), only 40.3% of caregivers of children under 5 years of age in northern Nigeria were aware of the malaria vaccine, whereas 91.9% expressed a willingness to accept it when available. Additionally, 89.7% were willing to pay for the vaccine. The study identified factors such as higher education levels, employment, prior experience with childhood vaccinations, and recent malaria episodes among caregivers as significant predictors of awareness. Recent investigations into vaccine safety concerns revealed that a substantial portion of vaccine hesitancy is also caused by believing in conspiracy theories [18] and misinformation spread by social media [19].

Despite these insights, there is limited evidence on caregivers’ awareness, perceptions, acceptance, and willingness to pay for the R21/MATRIX malaria vaccine in malaria-endemic communities in Lagos State, where population density, health-seeking behavior, and exposure to health information may differ from other states. Understanding these factors is critical for guiding effective vaccine rollout strategies in the region. This study aimed to assess the levels of awareness, perceptions, acceptance, and willingness to pay for the R21/MATRIX malaria vaccine among caregivers of children under five in endemic communities in Lagos, Nigeria, and to identify factors that predict these outcomes.

## Methods

### Study area

The study was conducted in Lagos, Nigeria, a major urban center characterized by high population density, with an estimated population of over 20 million people as of 2023 (World Bank, 2023). This diverse city serves as Nigeria's economic hub and is characterized by various cultures, ethnicities, and socioeconomic backgrounds. The state is divided into 20 local government areas and 37 local council development areas. Yoruba is the predominant spoken language, although English is the official language because of its cosmopolitan nature. Lagos State is Nigeria's commercial center, which houses major financial institutions, industries, and the country's largest seaports. The high prevalence of malaria in the region is explained by the risks factors from poor housing, rainy season breeding sites, population density, and proximity to water bodies, disproportionately affecting pregnant women and children.

### Study design

This was a community-based cross-sectional survey consisting of structured questionnaires administered to caregivers of children under five years of age in selected endemic communities in Lagos State.

### Inclusion and exclusion criteria

The study included primary caregivers of children under 5 years old, residents of the selected communities for at least 6 months, and those with the ability to provide informed consent. The exclusion criteria included caregivers under 18 years of age, women above 18 years without under 5 children, those with cognitive impairments preventing informed consent, and nonresidents or temporary visitors to the community.

### Sample size determination

The required sample size for this study was estimated via the Leslie–Kish formula for a single proportion.

$$\frac{{Z^{2} p(1 - p)}}{{d^{2} }}$$

where:

$n$= minimum sample size.

$Z$= standard normal deviate at 95% confidence level (1.96).

$p$= estimated proportion of caregivers aware of the malaria vaccine.

$d$= desired precision or margin of error.

Assuming a 95% level of confidence, a proportion of respondents aware of the malaria vaccine at 26% (2025), 5% precision [18] and a 10% anticipated nonresponse rate, the minimum sample size was 326 caregivers after.

### Sampling technique

A multistage sampling technique was employed for this study to balance focus on high-risk communities with representativeness at the household level. In the first stage, five malaria-endemic communities were purposively selected from different Local Government Areas (LGAs) in Lagos State based on documented high malaria prevalence. This selection at the household level minimized bias and improved the reliability of responses. The 5 endemic communities selected are Abule-Ijesha, Ikorodu, Badagry, Bariga, and Makoko, due to high prevalence of malaria in those regions [3]. Eligible households (those with children under 5 years of age) were identified via household lists obtained from community leaders. In the final sampling stage, Probability Proportional to Size (PPS) allocated households based on the number of eligible households in each community. Household lists from community leaders served as the sampling frame. Within each community, households were randomly selected, and one eligible caregiver per household was recruited until the community-specific sample size was reached.

### Study instrument

A pretested, semistructured, interviewer-administered, forty-item questionnaire was used to elicit information from the respondents. The questionnaire was developed by the researchers based on a review of existing literature on malaria prevention, malaria vaccine awareness, perception, acceptability, and willingness to pay, with items tailored to the R21/MATRIX malaria vaccine and the study context.

The questionnaire was divided into four sections: (i) respondents' sociodemographic characteristics, sex, age, marital status, ethnicity, religion, education level, employment status, monthly household income, and settlement type; (ii) knowledge and awareness of malaria and the R21/MATRIX vaccine; (iii) perceptions of the R21/MATRIX vaccine; and (iv) willingness to pay and acceptability.

The questionnaire was reviewed by experts in public health and malaria control to assess face and content validity, focusing on the relevance, clarity, and appropriateness of the items for the target population. Revisions were made based on their recommendations. The instrument was pretested among caregivers of under-five children in a malaria-endemic community outside the study area, and minor modifications were implemented to improve clarity and comprehension before the final administration.

### Data collection

The participants were given a detailed explanation of the study's nature. Informed consent was subsequently obtained from each respondent, and they were assured of confidentiality. Data were collected in person using printed questionnaires, which were given to participants to complete in hard copy. Trained research assistants provided guidance when needed. The questionnaire was translated into Yoruba, the local language. The English or Yoruba versions were used, depending on the respondent’s preference. Responses from the completed hard copies were subsequently entered into Microsoft Excel for analysis. The electronic dataset was stored on a password-protected computer accessible only to the research team. Regular backups were made to an external hard drive to prevent data loss. All files were encrypted, and identifiers were removed to ensure confidentiality and secure retrieval of data throughout the study.

### Data management/analysis

STATA was used to analyze the data obtained. Prior to analysis, the dataset was checked for completeness and consistency. Variables with missing values less than 5% were analyzed using complete case analysis. For variables with 5–10% missing, missing values were imputed using the mode for categorical variables and the median for continuous variables. Variables with more than 10% missing data, affected records were excluded from the specific analyses. Descriptive statistics were computed via summary statistics such as frequencies, means, percentages, and standard deviations. The relationships between the independent variables and dependent variables were assessed via binary logistic regression. To control for potential confounders, all significant variables were considered for multivariable logistic regression, which was applied to determine the independent effect of each variable on the outcome variables.

The Lagos State Health Research Ethics Committee granted ethical approval of the study protocol, including the instruments. Permission to carry out the study was obtained from the local government authority and head of household.

## Results

### Sociodemographic characteristics of the respondents

A total of 372 participants participated in the study, with diverse sociodemographic characteristics (Table 1). Most participants were female (90.1%) and aged 25–27 years (50.3%). The majority were married (87.6%) and identified as Yoruba (68.3%). Most participants were Christian (62.9%) or Muslim (36.6%). In terms of education, nearly half (49.2%) had secondary education, 45.4% had tertiary education, and 5.4% had no formal education. Over half (55.7%) were professionals or civil servants. The majority (63.4%) reported monthly household incomes between ₦50,000 and ₦100,000, with 25.3% below ₦50,000 and 11.3% between ₦101,000 and ₦200,000. Nearly all the participants resided in urban areas (96.8%), according to the National Population Commission’s (NPC) Enumeration Area (EA) classification, which categorizes communities as urban or rural based on population density, infrastructure, and settlement patterns. Most (89.5%) identified as parents of children under five, with 10.5% as guardians. A large majority (85.2%) had one child under five years of age.

**Table 1:** Sociodemographic characteristics of the participants, N = 372

Characteristics	Frequency, n	Percentage, %
Gender		
Male	37	9.9
Female	335	90.1
Age category		
20–24	85	22.8
25–27	187	50.3
28 +	100	26.9
Marital status		
Single	46	12.4
Married	326	87.6
Ethnicity		
Yoruba	254	68.3
Hausa	50	13.4
Igbo	66	17.7
Fulani	2	0.5
Religion		
Muslim	136	36.6
Christianity	234	62.9
Traditional	2	0.5
Education level		
No education	20	5.4
Secondary	183	49.2
Tertiary	169	45.4
Employment category		
Low-skilled/unemployed	23	6.2
Trader	142	38.2
Professional/civil servant	207	55.7
Monthly household income		
Below ₦50,000	94	25.3
₦50,000–₦100,000	236	63.4
₦101,000–₦200,000	42	11.3
Settlement type		
Rural	12	3.2
Urban	360	96.8
Relationship with children under five		
Parent	333	89.5
Guardian	39	10.5
Number of children under five		
1	317	85.2
2 or more	55	14.8

### knowledge and awareness of malaria and the R21/MATRIX vaccine

Only 9.7% (n = 36) of the participants had previously heard of the R21/MATRIX malaria vaccine, primarily through the internet (9.4%), with minimal awareness through TV (0.3%). In terms of self-rated malaria knowledge, nearly half (46.8%) rated their knowledge as high, 29.8% as low, and 23.4% as moderate. Most participants (83.3%) correctly identified mosquito bites as the primary mode of malaria transmission, while 15.9% mistakenly believed it was due to contaminated water, and 0.8% thought it was airborne. When asked about their awareness of other malaria vaccines or treatments, 38.2% of the participants responded affirmatively, whereas 61.8% were unaware of any other options (Table 2).

**Table 2:** Knowledge and awareness of malaria and the R21/MATRIX vaccine, N = 372

Variable	Frequency, n	Percentage, %
Have you heard about the R21/MATRIX malaria vaccine before today?		
Yes	36	9.7
No	336	90.3
If you answered 'Yes' to the previous question, how did you hear about it?		
Internet	35	9.4
TV	1	0.3
Self-rated knowledge of malaria		
Low knowledge	111	29.8
Moderate knowledge	87	23.4
High knowledge	174	46.8
What do you believe is the primary mode of transmission of malaria?		
Mosquito bites	310	83.3
Contaminated water	59	15.9
Airborne	3	0.8
Are you aware of any other malaria vaccines or treatments?		
Yes	142	38.2
No	230	61.8

### Associations between awareness of the R21/MATRIX malaria vaccine and selected sociodemographic characteristics of the participants

Compared with females, male participants were significantly more likely to be aware of the vaccine, with an odds ratio (OR) of 8.66 (95% CI: 3.92–19.13, p < 0.001). With respect to age, participants aged 25–27 years had lower odds of awareness (OR: 0.29, 95% CI: 0.13–0.67, p = 0.003) than those aged 20–24 years did, whereas the difference for those aged 28 + years was not statistically significant (OR: 0.52, 95% CI: 0.22–1.22, p = 0.134). Marital status was significantly associated, with married participants being less likely to be aware of the vaccine than single participants were (OR: 0.19, 95% CI: 0.08–0.42, p < 0.001). Education level was not significantly associated with awareness; participants with secondary education (OR: 0.87, 95% CI: 0.10–7.32, p = 0.897) and tertiary education (OR: 3.61, 95% CI: 0.46–28.1, p = 0.220) showed no significant differences in awareness compared with those with no formal education.

Moreover, employment status was not significantly associated with vaccine awareness. Employment as a trader (OR: 0.31, 95% CI: 0.03–3.61, p = 0.353) or as a professional/civil servant (OR: 4.17, 95% CI: 0.54–32.03, p = 0.170) did not significantly differ in awareness levels compared with low-skilled or unemployed participants. Compared with rural residents, urban residents were less likely to be aware of the vaccine, although this association was not statistically significant (OR: 0.30, 95% CI: 0.08–1.17; p = 0.084). In terms of relationships with children under five years of age, guardians had significantly greater odds of being aware than parents did (OR: 7.92, 95% CI: 3.61–17.34, p < 0.001). However, the number of children under five years of age was not significantly associated with awareness; having two or more children was not significantly different from having one child (OR: 0.50, 95% CI: 0.15–1.68; p = 0.260).

These results indicate that sex, age, marital status, and relationships with children under five years of age were significantly associated with vaccine awareness, with males, younger adults (20–24 years), single parents, and guardians being more likely to be aware of the R21/MATRIX malaria vaccine (Table 3).

**Table 3:** Association between awareness of the R21/MATRIX malaria vaccine and selected sociodemographic characteristics of the participants

Characteristics	Category	Odd Ratio, 95% CI, P value
Gender		
	Male	8.66 (3.92–19.13), p < 0.001
	Female	Ref
Age category		
	20–24	Ref
	25–27	0.29 (0.13–0.67), p = 0.003
	28 +	0.52 (0.22–1.22), p = 0.134
Marital status		
	Single	Ref
	Married	0.19 (0.08–0.42), p < 0.001
Education level		
	No education	Ref
	Secondary	0.87 (0.10–7.32), p = 0.897
	Tertiary	3.61 (0.46–28.1), p = 0.220
Employment category		
	Low-skilled/unemployed	Ref
	Trader	0.31 (0.03–3.61), p = 0.353
	Professional/civil servant	4.17 (0.54–32.03), p = 0.170
Settlement type		
	Rural	Ref
	Urban	0.30 (0.08–1.17), p = 0.084
Relationship with children under five		
	Parent	Ref
	Guardian	7.92 (3.61–17.34), p < 0.001
Number of children under five		
	1	Ref
	2 or more	0.50 (0.15–1.68), p = 0.260

### Perception of the R21/MATRIX malaria vaccine among the participants

A strong majority of participants expressed positive perceptions regarding the vaccine's safety, efficacy, and recommendations. Nearly all participants (98.1%) believed that the vaccine was safe for children under 5 years of age, and 98.9% believed it was effective in preventing malaria. Similarly, 98.9% felt that the benefits of the vaccine outweigh any potential risks. The level of confidence in health authorities' recommendations was high, with 98.1% indicating confidence in the guidance provided on the R21/MATRIX vaccine. While there was some concern about possible side effects, as expressed by 98.4% of the participants, the willingness to recommend the vaccine remained high, with 99.7% willing to recommend it to other caregivers and 99.2% happy to recommend it more broadly. There was also strong support for making the vaccine mandatory for children under 5 years of age in endemic areas, with 98.9% in agreement. Finally, the availability of the vaccine in the community contributed to a sense of security for participants, with 99.7% reporting that its availability would make them feel more secure about their child’s health. These findings indicate overwhelmingly positive perceptions of the R21/MATRIX malaria vaccine among caregivers of young children in the study (Table 4).

**Table 4:** Perception of the R21/MATRIX malaria vaccine among the participants, N = 372

Perception Statement	Yes, n (%)	No, n (%)
I believe the R21/MATRIX Malaria Vaccine is safe for children under 5	365 (98.1)	7 (1.9)
The R21/MATRIX Malaria Vaccine is effective in preventing malaria	368 (98.9)	4 (1.1)
The benefits of the R21/MATRIX Malaria Vaccine outweigh any potential risks	368 (98.9)	4 (1.1)
I trust the health authorities’ recommendations regarding the R21/MATRIX Malaria Vaccine	365 (98.1)	7 (1.9)
I am concerned about possible side effects of the R21/MATRIX Malaria Vaccine	366 (98.4)	6 (1.6)
I would recommend the R21/MATRIX Malaria Vaccine to other caregivers of under5 children	371 (99.7)	1 (0.3)
I believe that the R21/MATRIX Malaria Vaccine should be mandatory for all children under 5 in endemic areas	368 (98.9)	4 (1.1)
I am happy to recommend the R21/MATRIX Malaria Vaccine to others	369 (99.2)	3 (0.8)
The availability of the R21/MATRIX Malaria Vaccine in my community would make me feel more secure about my child’s health	371 (99.7)	1 (0.3)

### Acceptability of the R21/MATRIX malaria vaccine among the participants

When asked about their likelihood of vaccinating their child with the vaccine, 76.6% of the participants indicated that they would "definitely" vaccinate, with another 20.2% responding that they would "probably" vaccinate. Only a small minority expressed reluctance, with 3.0% indicating that they "probably will not" vaccinate and 0.3% stating that they "definitely will not" vaccinate. The recommendation of a healthcare provider plays a significant role, as 76.9% of participants would "definitely" vaccinate if advised by their provider, and 22.0% would "probably" vaccinate under the same circumstances. Cost was also an important consideration, with 38.2% reporting it as "extremely important" and 30.9% as "very important" in their decision-making process. Effectiveness data specific to Nigeria was another influential factor, with 40.1% stating that it would "greatly influence" their decision and 58.3% saying that it would "somewhat influence" it. Minimal side effects also had a strong effect on participants' willingness, with 23.7% stating that they would "strongly influence" their decision and 73.4% reporting that they would "somewhat influence" their choice. These results indicate a high level of acceptability of the R21/MATRIX malaria vaccine among caregivers, strongly influenced by healthcare provider recommendations, cost considerations, local effectiveness data, and minimal side effects (Table 5).

**Table 5:** Acceptability of the R21/MATRIX malaria vaccine among the participants, N = 372

Acceptability question	Frequency, n	Percentage, %
How likely are you to vaccinate your child with the R21/MATRIX malaria vaccine?		
Definitely will vaccinate	285	76.6
Most likely, will vaccinate	75	20.2
Unsure	0	0.0
Most likely, will not vaccinate	11	3.0
Definitely will not vaccinate	1	0.3
Would the recommendation of the vaccine by your child’s healthcare provider influence your decision to use it?		
Definitely yes	286	76.9
Most likely, yes	82	22.0
Unsure	3	0.8
Most likely, no	1	0.3
Definitely no	0	0.0
How important is the cost of the vaccine in deciding whether to vaccinate your child?		
Extremely important	142	38.2
Very important	115	30.9
Moderately important	25	6.7
Slightly important	47	12.6
Not important at all	43	11.6
Would information about the vaccine's effectiveness tested in Nigeria influence your decision to vaccinate?		
Greatly influence	149	40.1
Somewhat influence	217	58.3
Not influence	1	0.3
Unsure	5	1.3
Would knowing the R21/MATRIX Malaria Vaccine has minimal side effects influence your decision to vaccinate your child?		
No Influence	2	0.5
Unsure	9	2.4
Somewhat influence	273	73.4
Strongly influence	88	23.7

### Willingness to pay for the R21/MATRIX malaria vaccine among the participants

A majority (71.2%) of the participants expressed a willingness to pay, whereas 28.8% indicated that they would not pay for the vaccine. Among those willing to pay, 51.9% considered an amount between ₦1,001 and ₦5,000 as reasonable per dose. A total of 18.6% were willing to pay ₦1,000 or less, whereas only a very small percentage indicated that they would pay ₦5,001 to ₦10,000 (0.3%) or more than ₦10,000 (0.5%). When asked if knowledge of the vaccine’s high effectiveness and minimal side effects would increase their willingness to pay, 94.3% of the participants reported that this information would not change their willingness. In comparison, 5.7% stated that it would make them more willing to pay. These findings suggest that while a majority of participants are willing to pay for the vaccine, their willingness is limited to lower price ranges, and additional information about the vaccine’s effectiveness and safety does not significantly influence their decision (Table 6).

**Table 6:** Willingness to pay for the R21/MATRIX malaria vaccine among the participants, N = 372

Willingness to pay questions	Frequency, n	Percentage, %
Would you be willing to pay for the R21/MATRIX Malaria Vaccine if it is not provided for free?		
Yes	265	71.2
No	107	28.8
If you are willing to pay for the vaccine, how much would you consider a reasonable amount per dose?		
Not willing to pay	107	28.8
₦1000 or less	69	18.6
₦1001 to ₦5000	193	51.9
₦5000 to ₦10,000	1	0.3
More than ₦10,000	2	0.5
Would your be willing to pay for the vaccine change if you knew it was highly effective and had minimal side effects?		
Yes, I would be more willing to pay	21	5.7
No, my willingness to pay would not change	351	94.3

### Association between willingness to pay for the R21/MATRIX malaria vaccine and selected sociodemographic characteristics of the participants

Male participants were significantly more likely to be willing to pay than females were, with an odds ratio (OR) of 3.66 (95% CI: 1.26–10.61, p = 0.017). With respect to age, participants aged 25–27 years were less likely to be willing to pay than those aged 20–24 years were, although this difference was not statistically significant (OR: 0.59, 95% CI: 0.33–1.08; p = 0.087). There was also no significant difference for those aged 28 + years (OR: 0.78, 95% CI: 0.40–1.53, p = 0.467). Marital status had a significant effect, with married participants being less willing to pay than single participants (OR: 0.27, 95% CI: 0.10–0.70, p < 0.001). Education level also influenced willingness to pay, as participants with secondary education were less likely to be willing to pay than those with no education (OR: 0.20, 95% CI: 0.05–0.90, p = 0.035). However, no significant association was found for those with tertiary education (OR: 0.36, 95% CI: 0.08–1.61, p = 0.181). The employment category was not significantly associated with willingness to pay; both traders (OR: 0.65, 95% CI: 0.24–1.75, p = 0.394) and professionals/civil servants (OR: 1.08, 95% CI: 0.40–2.88, p = 0.879) showed no significant difference compared with low-skilled or unemployed participants.

Monthly household income was a significant factor. Participants earning ₦50,000—₦100,000 (OR: 2.49, 95% CI: 1.50–4.12, p < 0.001) and those earning ₦101,000—₦200,000 (OR: 2.48, 95% CI: 1.09–5.61, p = 0.030) were more likely to be willing to pay than those earning below ₦50,000.

There was no significant association between settlement type (urban vs. rural) and willingness to pay (OR: 0.82, 95% CI: 0.22–3.09, p = 0.770). Relationships with children under five years of age had a strong effect, with guardians showing significantly greater odds of willingness to pay than parents did (OR: 17.7, 95% CI: 2.40–131.0, p = 0.005). However, the number of children under five years of age was not significantly associated with willingness to pay (OR: 0.73, 95% CI: 0.40–1.34, p = 0.306). These results suggest that gender, marital status, education level, household income, and relationships with children under five significantly influence participants' willingness to pay for the vaccine (Table 7).

**Table 7:** Association between willingness to pay for the R21/MATRIX malaria vaccine and selected sociodemographic characteristics of the participants

Characteristics	Category	Odd Ratio, 95% CI, P value
Gender		
	Male	3.66 (1.26–10.61), p = 0.017
	Female	Reference
Age category		
	20–24	Reference
	25–27	0.59 (0.33–1.08), p = 0.087
	28 +	0.78 (0.40–1.53), p = 0.467
Marital status		
	Single	Reference
	Married	0.27 (0.10–0.70), p < 0.001
Education level		
	No education	Reference
	Secondary	0.20 (0.05–0.90), p = 0.035
	Tertiary	0.36 (0.08–1.61), p = 0.181
Employment category		
	Low-skilled/unemployed	Reference
	Trader	0.65 (0.24–1.75), p = 0.394
	Professional/civil servant	1.08 (0.40–2.88), p = 0.879
Monthly household income		
	Below ₦50,000	Reference
	₦50,000—₦100,000	2.49 (1.50–4.12), p < 0.001
	₦101,000 -₦200,000	2.48 (1.09–5.61), p = 0.030
Settlement type		
	Rural	Reference
	Urban	0.82 (0.22–3.09), p = 0.770
Relationship with children under five		
	Parent	Reference
	Guardian	17.7 (2.40–131.0), p = 0.005
Number of children under five		
	1	Reference
	2 or more	0.73 (0.40–1.34), p = 0.306

## Discussion

The R21/Matrix-M vaccine has displayed efficacy. It is the second malaria vaccine recommended by the WHO after the RST, S/ASO1, which was recommended in 2021 [20]. The impact of malaria on public health and its cost-effectiveness make it an efficient tool for controlling malaria in countries with high numbers of seasonal malaria cases and low- to middle-income countries. Understanding the factors that influence caregivers’ awareness level and willingness to accept vaccines plays a crucial role in informing policies and policymakers, healthcare professionals, and other social workers in areas that might need strengthening to promote vaccine acceptance and use [16].

The findings of this study indicate that awareness of the R21/MATRIX malaria vaccine among caregivers in Lagos was low (9.7%), despite high positive perception (98.1%) and acceptability (76.6%). This low level of awareness is consistent with findings from other Nigerian studies. For instance, Abdulkadir and Ajayi (2021) reported that only 20.1% of caregivers in Ibadan North had ever heard of a malaria vaccine [21], while Ajayi and Emeto (2021) found 40.3% awareness in Northern Nigeria [22]. Similarly, Musa-Booth et al. (2021) reported that approximately 30% of mothers in Abuja were aware of malaria vaccines, though only 4% had heard specifically of RTS,S [23]. In contrast, Chukwuocha et al. (2018) found a relatively higher awareness of a prospective malaria vaccine (48.2%) among caregivers in South Eastern Nigeria [24]. The consistently low awareness across most studies, including the present study, underscores the need for targeted education and social mobilization to improve knowledge of new malaria vaccines prior to implementation.

Despite low awareness, the perception and acceptability of the malaria vaccine were consistently high across studies. In this study, 98.1% of caregivers perceived the R21/MATRIX vaccine as safe and effective, and 76.6% expressed definite willingness to vaccinate their children. Comparable trends were reported by Chukwuocha et al. (2018), where 88.2% of respondents demonstrated a positive perception and 95.6% expressed intent to comply with vaccination [24]. Similarly, Musa-Booth et al. (2021) and Abdulkadir and Ajayi (2021) found positive attitudes of 91% and 87%, respectively, toward malaria vaccination, with high willingness to accept RTS,S or other vaccines [21, 23]. These findings indicate that even in the context of low prior knowledge, caregivers are generally receptive to malaria vaccines, suggesting that interventions focused on awareness could substantially enhance uptake.

Regarding factors influencing vaccine awareness and willingness to pay, this study found that gender, marital status, and caregiver relationship with the child were significantly associated with awareness, while higher household income and guardianship status predicted willingness to pay. These findings align with patterns observed in previous studies. Ajayi and Emeto reported that level of education, employment status, prior vaccination experience, and recent malaria infection significantly predicted awareness and acceptability [22]. Abdulkadir and Ajayi also highlighted the role of community health workers in influencing willingness to accept the vaccine [21]. Therefore, sociodemographic characteristics and prior exposure to health services consistently shape awareness, perception, and uptake intentions for malaria vaccines in Nigerian communities.

The participants expressed a positive perception of the R21/Matrix-M vaccine and a willingness to accept it. They believed that the vaccine would be safe for their children and that it would also be effective in malaria control. They, however, raised concerns over side effects, with the perspective of the benefit outweighing the potential risk. There is also a high level of trust in health authorities’ recommendations and guidance by such individuals. Caregivers were willing to recommend vaccines to other individuals and supported them in making R21/Matrix-M mandatory. This positive attitude can be linked to previous experience with vaccination programs and their benefits [24, 25]. These findings show that healthcare workers are crucial in raising awareness and perceptions among caregivers.

Despite the proven efficacy and cost effectiveness of vaccines, awareness among caregivers remains considerably low. Most of information about the vaccine came from the internet, whereas traditional mass media platforms, such as television and radio—which are historically effective in health promotion—were underutilized. Successful campaigns such as “Kick Polio Out of Nigeria” and the National AIDS and STD Control Programs demonstrate the transformative potential of mass media in reaching vulnerable populations [26, 27]. The Federal Ministry of Health should leverage these proven approaches to raise awareness of the R21/MATRIX vaccine, particularly through radio, which remains an effective medium for engaging vulnerable communities in remote regions.

Caregivers strongly positively perceived the vaccine, with 98.1% considering it safe and 98.9% believing in its effectiveness for malaria prevention. Vaccine acceptability was also high, with 76.6% of caregivers expressing a definite willingness to vaccinate their children. These findings highlight a readiness to adopt the vaccine if concerns about potential side effects are addressed through consistent, transparent communication. Trust in healthcare professionals and government authorities is a key enabler, underscoring the importance of mobilizing frontline healthcare workers to advocate for the vaccine at the grassroots level [28, 29].

Affordability, however, is a cause for concern. A total of 51.9% of caregivers were willing to pay between ₦1,001 and ₦5,000 for the vaccine. This emphasizes the urgent need for subsidies to ensure equitable access. Socioeconomic factors, including household income and caregivers' relationships with the child, significantly influence willingness to pay for the service [30]. Collaborative efforts among government agencies, donor organizations, and international partners are essential to reduce financial barriers and subsidize vaccine costs [31].

Maximizing the vaccine's impact will require a multifaceted approach, including culturally sensitive social and behavior change communication (SBCC) campaigns tailored to diverse populations, expanding the radio and television reach to disseminate accurate vaccine information, and addressing vaccine hesitancy through community engagement. The incorporation of the R21/MATRIX vaccine into routine immunization schedules and the alignment of these initiatives with the National Malaria Strategic Plan will further strengthen control efforts.

## Limitations

This study has several limitations. First, the willingness-to-pay (WTP) estimates may be subject to hypothetical bias, as respondents were asked to indicate the amount they would pay for the R21/MATRIX malaria vaccine without actual market exposure. Caregivers’ responses may therefore reflect perceived value rather than true affordability, potentially leading to overestimation or underestimation of actual WTP. Additionally, WTP can be influenced by respondents’ socioeconomic status, perceived risk of malaria, and prior knowledge, which may affect the generalizability of findings. Second, the cross-sectional design limits causal inference and only captures perceptions and intentions at a single point in time. Third, data were collected via self-report, which is subject to social desirability bias, particularly regarding vaccine acceptance. Finally, the study was conducted in selected communities in Lagos State, which may limit the generalizability of results to other regions with different sociocultural and economic contexts. Despite these limitations, the study provides valuable insights into caregivers’ awareness, perceptions, and potential adoption of the R21/MATRIX malaria vaccine.

## Conclusion

Awareness of the R21/MATRIX malaria vaccine among caregivers of under-five children in malaria-endemic communities in Lagos State was low; however, caregivers demonstrated overwhelmingly positive perceptions, high acceptability, and a moderate willingness to pay for the vaccine. These findings indicate that limited awareness, rather than negative attitudes, constitutes the primary barrier to potential vaccine uptake. Strengthening targeted community-based sensitization and health education initiatives that emphasize the safety, effectiveness, and benefits of the R21/MATRIX vaccine is therefore essential. By improving awareness, ensuring affordability, and reinforcing trust through healthcare providers and routine immunization platforms, the R21/MATRIX vaccine has the potential to significantly reduce malaria-related morbidity and mortality among children under five and contribute to Nigeria’s malaria elimination efforts.

## Data Availability

The data are available from the corresponding author upon reasonable request.

## Abbreviations

GAVI: Global Alliance for Vaccines and Immunization
GDP: Gross Domestic Product
IPTp: Intermittent preventive treatment in pregnancy
IRS: Indoor residual spraying
ITNs: Insecticide-treated nets
LSM: Larval source management
NCDC: Nigeria Centre for Disease Control
NEMC: Nigerian End Malaria Council
NMSP: National Malaria Strategic Plan
NPHCDA: National Primary Healthcare Development Agency
RTS, S/AS01: Recombinant Protein-Based Malaria Vaccine (RTS, S) with AS01 Adjuvant
R21/MATRIX-M: R21 Malaria Vaccine with Matrix-M Adjuvant
SBCC: Social and Behavior Change Communication
WHO: World Health Organization
